# Street smart: faster approach towards litter in urban areas by highly neophobic corvids and less fearful birds

**DOI:** 10.1016/j.anbehav.2016.03.029

**Published:** 2016-07

**Authors:** Alison L. Greggor, Nicola S. Clayton, Antony J.C. Fulford, Alex Thornton

**Affiliations:** a Department of Psychology, University of Cambridge, Cambridge, U.K.; b Department of Zoology, University of Cambridge, Cambridge, U.K.; c Centre for Ecology and Conservation, University of Exeter, Penryn Campus, Exeter, U.K.

**Keywords:** categorization, Corvidae, litter, object neophobia, urban gradient

## Abstract

The extent to which animals respond fearfully to novel stimuli may critically influence their ability to survive alongside humans. However, it is unclear whether the fear of novel objects, object neophobia, consistently varies in response to human disturbance. Where variation has been documented, it is unclear whether this variation is due to a change in fear towards specific novel stimuli, or whether it is symptomatic of a general change in fear behaviour. We measured levels of object neophobia in free-flying birds across urban and rural habitats, comparing corvids, a family known for being behaviourally flexible and innovative, with other urban-adapting bird species. Neophobic responses were measured in the presence of different types of objects that varied in their novelty, and were compared to behaviour during a baited control. Corvids were more neophobic than noncorvid species towards all object types, but their hesitancy abated after conspecifics approached in experimental conditions in which objects resembled items they may have experienced previously. Both sets of species were faster to approach objects made from human litter in urban than rural areas, potentially reflecting a category-specific reduction in fear based on experience. These results highlight species similarities in behavioural responses to human-dominated environments despite large differences in baseline neophobia.

## Highlights

• Corvids demonstrate higher object neophobia than other species.
• Corvids and other species are less fearful of litter in urban than rural areas.
• Urban and rural populations do not differ in general object neophobia.
• Adjusting to urban areas may cause specific, rather than general reductions in fear.

Animals' responses to novel stimuli may influence their survival as humans drastically alter habitats (Robertson, Rehage, & Sih, 2013). The extent to which animals respond fearfully to novelty (i.e. demonstrate neophobia) may help or hinder their success, depending on the dangers and benefits associated with novelty. For example, high levels of object neophobia may help animals avoid danger should the objects harbour predators or toxins, but reduced neophobia allows animals to approach and exploit potentially advantageous novel resources (Greenberg & Mettke-Hofmann, 2001). Since human-dominated habitats offer combinations of food, dangers and habitat types that differ substantially from less undisturbed environments, examining how animals respond behaviourally to novelty is important in understanding how they adjust to man-made changes in the environment (Greggor, Clayton, Phalan, & Thornton, 2014).

Urban areas exert strong selection pressures that often reduce species richness for vertebrate and invertebrate groups (McKinney, 2008). Although some bird species thrive in urban areas, no single defining trait predicts a species' urban presence (Croci et al., 2008, Kark et al., 2007, Møller, 2014, Shochat et al., 2006). Instead, success in urban environments may depend on species' ability to adjust to the demands of a new habitat by modifying behaviour, such as foraging strategies or the timing of breeding attempts (Kark et al., 2007, Shochat et al., 2006, Sol et al., 2002). Behavioural flexibility may be crucial in allowing animals to reduce costly and unnecessary fear responses or to increase them to deal with new dangers. For example, some urban birds are able to avoid investing in unnecessary antipredator responses by selectively responding to specific threatening humans (Davidson et al., 2015, Lee et al., 2011, Levey et al., 2009). However, it is unclear whether areas of human disturbance also favour selective reductions in fear towards other stimuli, such as potentially dangerous objects.

There is no consensus about the optimal level of object neophobia in urban environments because opposing hypotheses predict benefits for high or for low neophobia. Some studies suggest that less neophobic individuals are faster to interact with and solve novel foraging tasks (Benson-Amram and Holekamp, 2012, Biondi et al., 2010, Boogert et al., 2008, Griffin and Guez, 2014). Since human litter provides opportunities for foraging that requires the manipulation of novel objects, such as food packaging, reduced neophobia may make animals more likely to innovate with novel food or objects when invading novel habitats (Greenberg, 2003, Greenberg and Mettke-Hofmann, 2001, Martin and Fitzgerald, 2005). Accordingly, urban common mynas, *Acridotheres tristis*, have been shown to be less neophobic than suburban conspecifics (Sol, Griffin, Bartomeus, & Boyce, 2011), and urban groups of house sparrows, *Passer domesticus*, solve tasks more quickly than rural ones (Liker & Bókony, 2009). Such reductions towards fear-related stimuli in urban environments has been documented in other behaviours such as flight initiation distance (Clucas and Marzluff, 2012, Mccleery, 2009, Moller, 2010, Møller, 2008), a dampened corticosterone stress response (Grunst, Rotenberry, & Grunst, 2014) or both (Atwell et al., 2012) (but note that these stress hormone patterns are not universal, see Bonier, 2012).

In contrast, increased neophobia may be favoured in potentially dangerous locations where exploration may expose animals to threats such as generalist predators or poisons (Brown et al., 2013, Greenberg, 2003). Urban areas typically contain more of these threats (Evans et al., 2009, Sims et al., 2008, Sorace, 2002, Sorace and Gustin, 2009). Laboratory manipulations of predation pressure in fish show that individuals' predator neophobia can plastically respond to the dangers of the environment (Brown et al., 2013), and that experience with these pressures can increase survival upon reintroduction into the wild (Ferrari, Mccormick, Meekan, & Chivers, 2015). Additionally, urban environments may select for increased neophobia over time. Human commensal species of wild rats, for example, show higher levels of object neophobia than laboratory and feral strains that do not have a history of surviving alongside a rat poison (Cowan, 1977). Similarly, elevated levels of object avoidance have been documented in house sparrows and shiny cowbirds, *Molothrus bonariensis*, in urban compared to rural habitats (Echeverría & Vassallo, 2008).

Studies may have found conflicting relationships between neophobia and urban areas for several reasons. First, different species may respond in divergent ways to urban selection pressures. Interspecies comparisons between and within environments are crucial to explaining human impact on temperament traits, such as neophobia, but they are rarely conducted in the wild (Archard and Braithwaite, 2010, Réale et al., 2007). Second, studies often measure neophobia in subtly different ways. Tests must present objects that accurately represent either known or novel stimuli because avoidance should only be interpreted as neophobia if it reflects a response to novelty, rather than a generalized fear response (Greggor, Thornton, & Clayton, 2015). Third, neophobia tests are classically conducted on isolated individuals (e.g. Greenberg, 1990), yet the presence of foraging conspecifics is likely to influence novelty approach in groups in the wild. Therefore to assess wild birds' responses towards novelty and objects characteristic of urban and rural spaces, we compared behavioural responses of foraging groups towards several types of objects across a range of bird species.

We presented free-flying bird communities with an object made from either natural items that mirrored natural stimuli, litter items that mimicked anthropogenic foraging opportunities in urban areas, or entirely artificial objects designed not to resemble any familiar stimulus. We examined the responses of 12 species of urban-exploiting birds that ranged in size, foraging ecology and evolutionary history. Five of these species were corvids (Corvidae), a family often described as very neophobic (Greenberg and Mettke-Hofmann, 2001, Heinrich et al., 1995, Marzluff and Heinrich, 1991) yet highly innovative and skilled at exploiting novel opportunities (Emery and Clayton, 2004, Nicolakakis and Lefebvre, 2000), a seemingly paradoxical combination considering that neophobia is commonly thought to inhibit innovation (Greenberg, 2003, Griffin and Guez, 2014). To our knowledge corvid object neophobia has not been tested across urban gradients before, nor has their reputed high level of neophobia been verified through comparison with other wild species. We compared their neophobic responses to those of the other seven participating species to determine how universal urban neophobia changes might be. Both sets of species could, in theory, benefit equally from reduced neophobia in urban areas if it allowed for increased feeding opportunities around human-created packaging and waste. Corvids in urban areas have been reported to consume more human refuse than rural conspecifics (Rowley & Vestjens, 1973), and other bird species have been known to rely on anthropogenic food sources, especially during the winter (Orell, 1989). However, both sets of species also face potential dangers associated with the novelty they encounter, such as urban predators, including cats (Evans et al., 2009, Sims et al., 2008, Sorace, 2002, Sorace and Gustin, 2009). Therefore selectively avoiding certain types of objects, without having to relax their overall defences, would allow urban birds to take advantage of beneficial types of novelty. Additionally, since both the corvid and noncorvid groups contained social foraging species, known to make foraging decisions based on the behaviour of conspecifics (e.g. Aplin et al., 2012, Chiarati et al., 2012), the presence of conspecifics could help birds distinguish beneficial from dangerous novelty.

We predicted that: (1) corvids would show higher neophobia than noncorvids towards novel objects within habitats; (2) both sets of species would reduce their neophobic behaviour in urban areas towards objects that would be less novel there, such as litter in urban areas; and (3) foraging birds would be more likely to approach objects after a conspecific visited.

## Methods

Twelve feeding tables were set up across human population gradients in distinct geographical regions in the east and southwest of England (Cambridgeshire, eight tables; Cornwall, four). We estimated the extent of human presence in the vicinity of each table based on the amount of impervious surface cover, such as tarmac and rooftops, in the 1 km^2^ surrounding the site. Surface cover area was calculated by manually drawing polygons on satellite images using the land area calculator in Google Earth Pro. All table locations with surface cover higher than 20% were classified as high human impact zones (mean 45.6 ± 7.2%), less than 6% as low impact zones (mean 3.7 ± 0.5%; see Table 1). For clarity we refer to these areas as urban and rural, but acknowledge our areas with the highest impervious surface area are closer to the range commonly reported for suburban measures of cover (20–50%; Marzluff, 2001, McKinney, 2002, McKinney, 2008). Rural sites were on large plots of private land where litter was almost completely absent, while urban sites were located in public spaces or small gardens adjacent to busy streets. The two urban/rural gradients were located 430 km apart, ensuring that distinct communities of birds were surveyed. Corvids were colour-ringed in these areas as part of related study sites (Cambridgeshire: 323 jackdaws, *Corvus monedula*, three jays, *Garrulus glandarius*; Cornwall: 734 jackdaws, 79 rooks, *Corvus frugilegus*, eight crows, *Corvus corone*, six jays, six magpies, *Pica pica*). Data were collected on these ringed individuals, on all other unringed corvids and on the unringed individuals of seven species outside the corvid family (blue tit, *Cyanistes caeruleus*; great tit, *Parus major*; European robin, *Erithacus rubecula*; common blackbird, *Turdus merula*; common wood pigeon, *Columba palumbus*; common chaffinch, *Fringilla coelebs*; house sparrow) that foraged during our trials. Although all of the species that participated are known to live in both rural and urban areas, not all of them visited both urban and rural tables (see Appendix Table A1).

In the weeks leading up to the study, feeding tables were regularly baited between the hours of 0800 and 1400 with one cup of peanuts, to ensure that birds in the surrounding areas foraged readily at the tables. Tables were deemed ready for the experiment if a corvid and a noncorvid species took food from the table within 90 min of baiting for at least 3 days in a row. A total of 77 trials were run from late January through March during the winters of 2013 and 2014 (see Table A2). The Cambridgeshire gradient was sampled in both 2013 and 2014, the Cornish gradient in 2014 only. The trials fell outside the breeding season for all participating species, except for the rook, which commences breeding in March, but all participating birds were known to be independently foraging adults since trials took place before juvenile rooks fledged.

**Figure A1:**
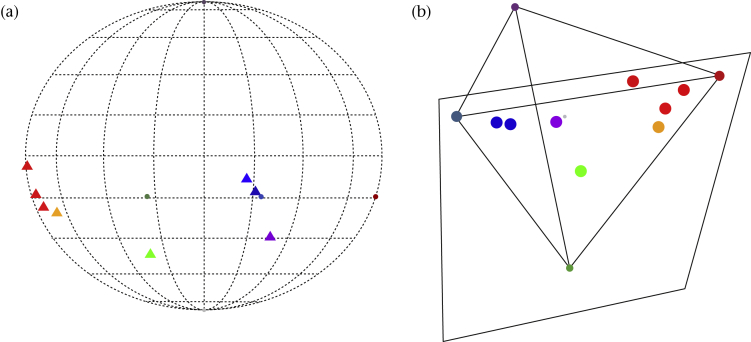
(a) Two-dimensional and (b) three-dimensional projection plots of materials used for novel objects, plotted in the avian tetrahedral visual space. Both plots show the range of material colours that were used. Material colours are represented by triangles in (a) and by central points in (b). Circles in (a) and triangle vertices in (b) provide reference points to the limit of visible wavelength for each receptor type. Only materials that occupied different visual spaces (such as the red and green triangles in (a)) were used in the same novel object.

**Figure 1:**
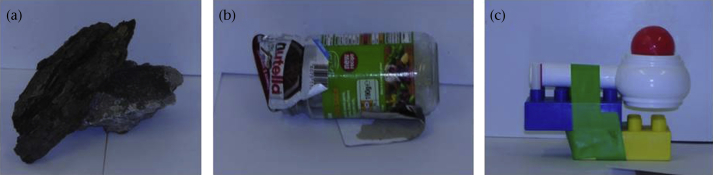
Examples of (a) Natural, (b) Litter and (c) Novel types of objects for each test condition.

Three separate classes of objects were used to assess the specificity of birds' fear responses within environments, i.e. to test whether they would respond neophobically to any new object placed on the feeding table, or respond less fearfully towards objects common in the surrounding habitat. Novel objects were built out of colourful, shiny, artificial materials that did not resemble any naturally occurring shape or animal, and did not have any parts that could resemble eyes. Materials used for novel object construction were determined to be distinctive to the birds via spectral analyses in the avian visual space (see Appendix for methods and Fig. A1 for plots). No two materials that were separated by less than one just noticeable difference (JNDs; values less than one JND are indistinguishable, Vorobyev & Osorio, 1998) were used in the same object. Litter objects were made from man-made food wrappers and containers (e.g. crisp bags, jam jars and Styrofoam fast-food containers) and were designed to mirror stimuli commonly found in urban areas. Natural objects consisted of rocks, leaves and sticks found in the local area (see Fig. 1 for examples of object types). The objects from all conditions were of similar size: about half of the volume of a jay, the smallest corvid in our study. No object was repeated at any one table, but the same objects were used on urban and rural tables so any comparisons between the populations would be towards the same objects. Although few ringed individuals were seen at multiple tables (*N* = 12), no individual was seen at multiple tables when the same object was presented. To ensure we reliably measured fear, as opposed to exploration or food motivation, we compared neophobic behaviour with behaviour during a control condition in which there was food but no object on the table (Greggor et al., 2015, Mettke-Hofmann et al., 2013).

In all object conditions, an object was placed on the same corner of the table, and one cup of peanuts placed in the centre. In the control condition, food was placed on the table alone. One cup contained about 320 peanuts, several times more than what a single individual of our largest participating species could consume. Trials lasted 90 min or until all of the food was consumed, whichever came first. All four conditions (Novel, Litter, Natural and Control) took place on consecutive days, at the same time of day. The order of conditions was determined for each table with an online random number generator. In attempts to create an even number of trials across regions, several tables had additional sets of trials on following days and were given a different time of day for each set. Trials were video recorded using a Panasonic HDC-SD90 camcorder, wrapped in camouflage tape, from a tripod placed approximately 10 m away and from the same location at each table for all trials.

Videos were subsequently analysed with Observer XT (Version 7.0, Noldus Information Technology, Wageningen, The Netherlands), to record the timing of each bird's visit, the amount of food it ate, its species and, where applicable, its colour ring combination. Fourteen full trials were coded by two people, one of whom was blind to the experimental questions.

### Ethical Note

This work was carried out under Home Office licence (PIL 70/25311, PPL to A.T. 80/2371) and in accordance with the ASAB Guidelines for the Treatment of Animals in Behavioural Research and Teaching. Birds were ringed under licence from the British Trust for Ornithology (no. C6079, C5752, C5746), either as nestlings in previous years (all jackdaw nestlings in the population are ringed) or as adults using ladder traps and nestbox trap doors.

### Analysis

We analysed four response variables, clustered into two sets of analyses. The first set allowed us to test whether or not corvids were more neophobic than the other set of species, and whether or not urban and rural populations of these groups differed. Specifically we tested these hypotheses by investigating the behaviour of the least neophobic bird of each species by measuring (1) whether any member of that species (either ringed or unringed) appeared at the table during the trial, and (2) their latency of arrival from the time of table baiting. The second set focused on a restricted data set, excluding bird species that did not appear more than once over the course of the trial, to analyse whether birds behave differently towards the types of objects after a conspecific had visited the table. Therefore we investigated the (1) feeding rate and (2) visit rate of birds after the first conspecific had foraged. Each set of analyses investigated the influence of the following main explanatory terms: experimental condition (Control, Natural, Litter or Novel), habitat (Urban or Rural), species group and interactions between these factors. They all controlled for the potential confounding variables of date, region (Cambridgeshire or Cornwall), time of day, year of experiment and the presence of other bird species where necessary (i.e. adding a binary variable that denoted whether another bird from their species group had arrived before them).

#### Least neophobic individuals: appearance at tables

In contrast to laboratory studies that can force interactions with novelty, wild animals can respond by avoiding novelty entirely (Greenberg, 2003). To determine the factors influencing whether or not birds appeared at tables, we ran a generalized linear mixed model (GLMM) with a binomial error structure (Appeared = 1, Did not appear = 0). Only the first observation of each species was used, with each species at a single trial counting as one data point. All potentially confounding variables (date, region, time of day, year) were included as covariates. Feeding table and experimental trial were assigned as random factors to account for repeated measures from the same table and from the same 90 min trial. Additionally, species was included as a random factor to control for differences between species within each species group (Corvids; Noncorvids). Species that were never observed during any trial at a given table, nor seen in the surrounding habitat during field observations, were removed from analysis at that table. Analysis started with a maximal model, which was simplified through backwards stepwise elimination. Terms were kept if their exclusion increased the model's Akaike's information criterion (AIC) value by at least two. Model selection is detailed in Table A3. Once a minimal model was determined, *P* values and effect sizes were calculated for each remaining covariate, and listed in the text (Zuur, Ieno, Walker, Saveliev, & Smith, 2009). Model assumptions were validated through inspection of diagnostic plots.

#### Least neophobic individuals: approach latency

Since approach latency is a commonly used measure of neophobia (Mettke-Hofmann, Rowe, Hayden, & Canoine, 2006), we examined how long it took for the first individual of each species to arrive at the table following baiting. To account for the fact some species may have arrived if given more time, we ran a Cox proportional hazards regression model (see Bókony, Kulcsár, Tóth, & Liker, 2012), on the same variables of interest, and potential confounding covariates as the GLMM. We clustered the observations around Trial, Species and Table, to account for interdependence in the data. The potential influence of other bird species on arrival time was accounted for by adding two binary terms: one denoted whether a corvid had arrived before the current observation, the other noting whether a noncorvid had arrived beforehand.

#### Group responses: feeding and visit rate

Many individuals had the opportunity to forage after potential conspecific social cues were available because trials offered hundreds of peanuts. We analysed each species' feeding rate and visit rate to assess whether birds continued to avoid objects after a conspecific had foraged at the table. Each peanut picked up from the table counted as one food piece. A visit was defined as a bird touching the feeding table. Total numbers of food pieces and visits were calculated from the behaviour of the second bird through to the end of the trial. Both rates were calculated by dividing the food pieces and visit totals by the number of minutes from the first visitor to the end of the trial. Both food and visit rate data were non-normal, so were log transformed and analysed with separate linear mixed models (LMMs), using the same explanatory variables, random effects and model selection methods as the appearance at tables GLMM.

All statistics were conducted in R (R Core Team, 2015), and models were created using the lme4 or survival package (Bates, Maechler, Bolker, & Walker, 2013).

## Results

In total we recorded 4300 visits and the consumption of 15 245 pieces of food across the 77 trials. Five species of corvid and seven species from other bird families participated in the experiment, with considerable variation in the species assemblages and visit numbers at each table (Appendix Table A1). Overall, the presence of corvids at the tables did not deter the other bird species from foraging, as corvid visits were often very short (<2 s), allowing plenty of time within the 90 min for other bird species to visit.

Intercoder reliability was perfect for species appearance (Cohen's kappa = 1.0), and extremely high for arrival time (one-way intraclass correlation coefficient: ICC = 0.99), visit number (ICC = 0.99) and the amount of food eaten (ICC = 0.96). All results reported below are derived from data that included all birds, regardless of whether or not they were ringed. The subset of data containing only ringed corvids indicates that the main appearance and arrival time results below do not depend on the behaviour of just a few individuals (see Appendix). Additionally, the effects discussed below were also present when analyses were conducted only on data from the two species from each group that visited the most (jackdaws/rooks and blue tits/great tits; Appendix Tables A4, A5).

### Table Appearance

**Figure 2:**
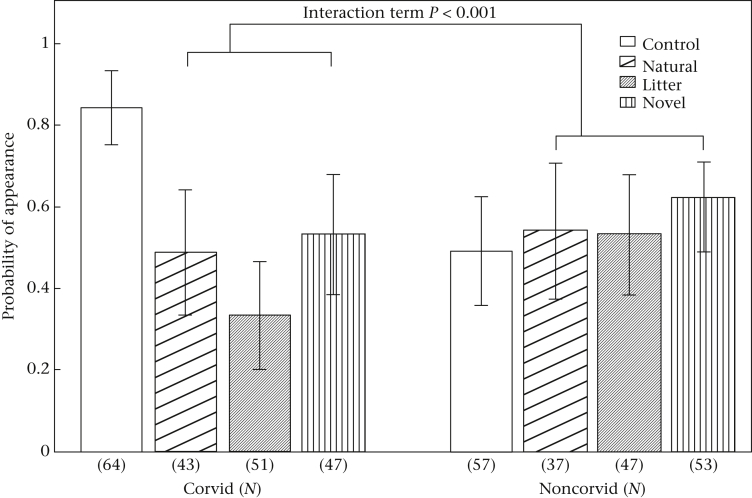
Interaction between species group (Corvid versus Noncorvid) and conditions. The control condition served as the reference category for all object conditions, and noncorvids for species group. GLMM: *N* = 399 observations, Corvid*Natural, estimate + SE = −2.67 + 0.80, *z* = −3.34, *P* < 0.001; Corvid*Litter, estimate + SE = −3.79 + 0.79, *z* = −4.78, *P* < 0.001; Corvid*Novel, estimate + SE = −3.00 + 0.77, *z* = −3.89, *P* < 0.001. Bars show means from raw data ± SE. Sample sizes (in parentheses) reflect number of observations; each species at each trial is one observation.

Corvids and noncorvids responded differently to the experimental conditions in their probability of appearing at the tables. Overall, there was an interaction between species group and response towards the objects: corvids were less likely to appear at tables when any type of object was present than in controls when no object was present, while we found no evidence that noncorvid species differed in appearance across any condition (see Fig. 2 for interaction details). Additionally, all birds were statistically more likely to appear as the date progressed, but the effect size was very small (GLMM: *N* = 399 observations, estimate + SE = 0.02 + 0.01, *z* = 2.54, *P* = 0.011). Birds were equally likely to appear at tables in urban and rural areas, and none of the other potential confounding variables were retained in the final model (see Table A3).

### Arrival Latency

**Figure 3:**
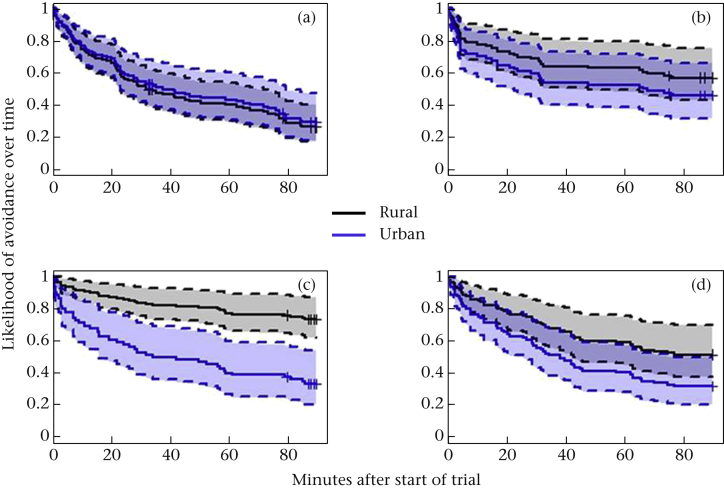
Survival curves showing the probability of not arriving over time, broken down by habitat and condition. Solid lines show survival curves, dotted lines indicate 95% confidence intervals. Black lines and shading plot data from rural areas, blue lines and shading from urban areas. Control conditions (a) served as the reference category for object comparisons, and rural areas for the urban gradient. Cox proportional hazards regression: *N* = 399 observations, 233 events, (b) Urban*Natural, rho = 0.045, χ^2^ = 0.70, *P* = 0.402; (c) Urban*Litter, rho = −0.243, χ^2^ = 21.61, *P* < 0.001; (d) Urban*Novel, rho = −0.055, χ^2^ = 0.64, *P* = 0.425.

Birds arrived faster in urban than rural areas, but only in Litter conditions (see Fig. 3). Additionally, corvids arrived more slowly than noncorvids (rho = 0.170, χ^2^ = 8.75, *P* = 0.003). Finally, birds in Cornwall arrived slightly slower than in Cambridgeshire (see Table 2, Appendix Table A6), and earlier in the morning birds arrived slightly faster than later in the day (rho = −0.126, χ^2^ = 3.92, *P* = 0.048; see Table 2, Appendix Table A6).

### Group Measures: Food Consumption and Visit Rate

**Figure 4:**
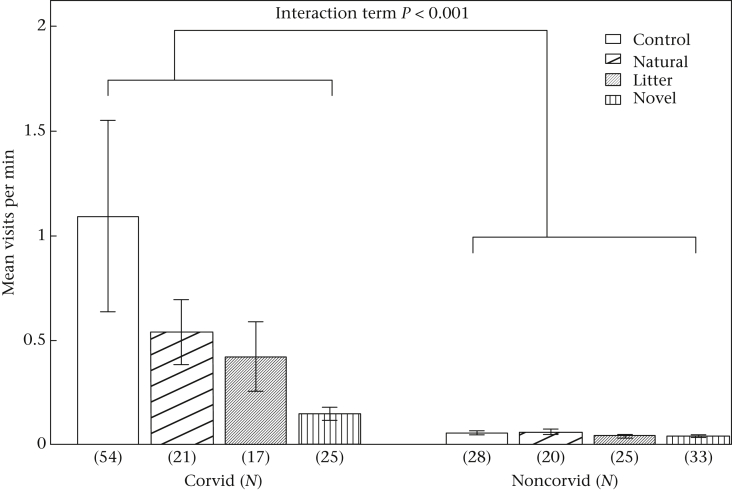
Interaction between species group (Corvid versus Noncorvid) and novel object condition in visit rates. The control condition served as the reference category for all object conditions, and noncorvids for species group. LMM, *N* = 176, Corvid*Natural, estimate + SE = 0.02 + 0.43, *z* = 0.04, *P* = 0.967; Corvid*Litter, estimate + SE = −0.58 + 0.43, *z* = −1.33, *P* = 0.183; Corvid*Novel, estimate + SE = 1.18 + 0.43, *z* = −2.72, *P* = 0.007. Bars show means from raw data ± SE.

**Figure 5:**
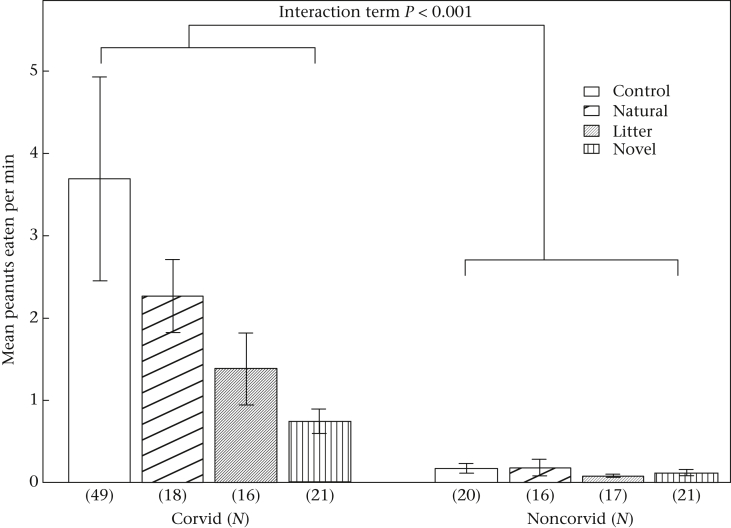
Interaction between species group (Corvid versus Noncorvid) and Novel object condition in feeding rates. The control condition served as the reference category for all object conditions, and noncorvids for species group. LMM, *N* = 178, Corvid*Natural, estimate + SE = 0.15 + 0.46, *z* = 0.32, *P* = 0.750; Corvid*Litter, estimate + SE = −0.46 + 0.47, *z* = −0.98, *P* = 0.325; Corvid*Novel, estimate + SE = −1.36 + 0.40, *z* = −3.40, *P* < 0.001. Bars show means from raw data ± SE.

Both the food consumption and visit rate models showed a similar interaction between species group and condition. Corvid species had lower feeding and visit rates in novel object trials than in control trials, while noncorvid species fed and visited at similar rates across all conditions after a conspecific had foraged (see Figure 4, Figure 5). Additionally, all species showed increasing visit and feeding rates as the dates progressed, but the effect sizes were very small (Feeding: estimate + SE = 0.01 + 0.005, *z* = 2.67, *P* = 0.008; Visit: estimate + SE = 0.01 + 0.005, *z* = 2.67, *P* = 0.008). Feeding and visit rates were similar across urban and rural habitats, and no other factors had significant effects in the model (see Table A3).

## Discussion

Although behavioural plasticity is commonly considered to be vital in allowing some species to survive in novel environments (Sol et al., 2002), it is unclear whether plasticity in fear around novelty is due to a general or specific modification of fear. In contrast to some previous studies (Bókony et al., 2012, Sol et al., 2011) we did not find reduced neophobia in urban birds, as responses towards novel objects were similar across habitats. However, as both species groups arrived faster around litter objects in urban than rural areas, their behaviour potentially reflects a specific reduction in fear towards a commonly occurring type of object. These patterns emerged despite the fact that corvid and noncorvid species differed in their neophobic responses and in their behaviour after the first individual foraged. Corvids appeared markedly more neophobic than other species in avoiding tables with any type of object, but were selective in how they responded to object types after a conspecific had foraged, only eating and visiting less around novel objects. Therefore our results indicate that both sets of species adjusted to urban areas by reducing fear towards regularly encountered objects despite both expressing different levels of fear.

Urban bird populations arrived faster than rural populations when the litter objects were present on tables. This result indicates that instead of showing generalized, population level reductions in neophobia, urban birds expressed a lower level of fear only towards specific, potentially rewarding objects. Such specific differentiation between litter and novel objects would be unlikely to have arisen through genetic change alone because the two types of objects share many perceptual features. Therefore the population differences are more likely to reflect learned categorization as a result of different experience. Through repeated exposure to anthropogenic objects, birds may have been able to better distinguish between them and other types of novelty because as exposure to stimuli increases so does the ability to differentiate their details (Hall and Honey, 1989, Shettleworth, 2010). Better abilities to differentiate man-made objects, and continued rewards around objects made of litter, would encourage birds to form a category of litter objects that shared some common stimuli. Whether birds' flexibility in mediating fear towards litter versus other types of objects is simply due to an increased exposure to stimuli (e.g. Lee et al., 2011, in differentiating humans), or whether urban and rural birds differ in their bias to flexibly classify stimuli may be important in determining whether success of urban-exploiting species is a result of behavioural adjustments.

While corvids and noncorvids responded similarly to litter objects, corvids were overall more neophobic than other bird species. Corvids appeared at tables less often during all object conditions than to controls, while other bird species were not deterred by the presence of objects, confirming suggestions that corvids are neophobic as adults (Greenberg and Mettke-Hofmann, 2001, Heinrich, 1988). Indeed, corvids' sensitivity to novelty was so pronounced that the presence of new objects on familiar feeding tables, even when these objects were natural materials that they may have encountered every day, reduced their probability of visiting tables relative to controls. Although the link between object neophobia and predatory wariness is unclear (Carter, Marshall, Heinsohn, & Cowlishaw, 2012), we speculate that human behaviour towards the species groups in this study may differ in ways that may help explain the comparatively high levels of corvid fear. Human discouragement in the form of chasing, shooting or threatening unpopular species has been shown to increase the fear responses of targeted birds towards humans (Clucas & Marzluff, 2012). In the U.K., humans actively encourage smaller songbirds to forage in their gardens, as 60% of households with gardens provide food for wild birds (Department of the Environment, 2002). In contrast, corvid species often face persecution by people because they are classified as vermin under U.K. law (Wildlife and Countryside Act 1981) and are targeted by deterrents and culling efforts (Henderson, 2002). While persecution may be higher in rural areas, it still occurs in urban populations; urban jackdaws, for instance, come into conflict with people since they often nest in chimneys (Röell, 1978, Salvati, 2002).

Despite their persecution, corvids' high level of neophobia may be seen as paradoxical because they are also known for their high rates of behavioural innovation (Emery and Clayton, 2004, Nicolakakis and Lefebvre, 2000): two traits that do not normally correlate (Greenberg, 2003). The mechanism through which their neophobia subsides to allow them to manipulate objects and solve problems is unknown, but potentially they are able to rapidly learn to categorize novelty as ‘safe’ or ‘unsafe’, similarly to how they can categorize other specific threatening stimuli, such as dangerous humans (Davidson et al., 2015, Lee et al., 2011, Marzluff et al., 2010), or known versus unknown predators (Marzluff, Delap, & Haycock, 2015). Whereas corvids fed and visited at equal rates to the control condition when social cues were available around natural and litter objects, these rates were significantly reduced around novel objects. This suggests that corvids may have classified objects according to their degree of novelty with the aid of social cues, with ‘less novel’ treated as ‘safer’. This type of flexibility in responding to object types may explain how corvids can be so neophobic, but also highly innovative around objects with which they may have prior experience. However, this ability is clearly not unique to corvids, as the other bird species that participated in this study also showed differentiation between certain types of objects in responding less fearfully towards litter than novel objects in urban populations. The extent to which novelty categories and social cues influence corvids' neophobic behaviour deserves future research if their behavioural adaptation to human-altered environments is to be better understood. Specifically, it is yet to be established whether or not species with greater opportunities for social learning due to their social system are more likely to use social cues around novelty.

As part of the suite of behaviours that can change with human disturbance, understanding where and why neophobia levels differ could be of great importance in conservation and wildlife management contexts (Greggor et al., 2014). We demonstrated that species respond similarly to experience in areas of human disturbance, despite exhibiting different levels of neophobia. However, it remains unclear how much exposure to objects is needed before animals no longer categorize stimuli as novel and thus fear-inducing. Future work is needed to reveal how population-specific patterns of object avoidance emerge in urban areas. Studies that examine the ontogeny of neophobic behaviours in urban versus rural areas could be particularly informative in investigating the role of individual experience in driving neophobia and other behaviours. Additionally, research testing how animals learn to distinguish ‘safe’ versus ‘unsafe’ object categories may help us understand the processes behind behavioural adjustments to urban areas. Together these investigations may explain why certain species and not others are able to behaviourally adjust and thrive in human-dominated environments.

## Figures and Tables

**Table A1:** Species participation at feeding tables

		Cambridge								Cornwall			
Rural							Urban	Rural	Urban		
B	D	H	I	K	M	N	J	PF	PHD	PHS	SC
Corvid	Carrion crow *Corvus corone*	5	1	13	22		2						
Eurasian jay *Garrulus glandarius*	38	115	7	130	105	110	82					69
Eurasian magpie *Pica pica*	1					9	1			7		
Jackdaw *Corvus monedula*	40	31	173	138	20	44		238	1161	82	26	968
Rook *Corvus frugilegus*	1		54	11	3	1						206
Noncorvid	Blue tit *Cyanistes caeruleus*	4	17		23	1	6		56		20	5	38
Common blackbird *Turdus merula*									1		5	14
Common chaffinch *Fringilla coelebs*	1								26			5
Common woodpigeon *Columba palumbus*								22				
European robin *Erithacus rubecula*									21			22
Great tit *Parus major*	11	2	12	28		1		1	10		4	35
House sparrow *Passer domesticus*									13			

Numbers indicate their number of visits at each table across all conditions.

**Table A2:** Number of trials per condition and table

Region	Condition						Total
	Table	Control	Rubbish	Natural	Novel
Cambridgeshire	Rural	B	1	2	1	1	5
D	1	1	1	1	4
H	3	1	1	1	6
I	2	1	1	1	5
K	1	1	1	1	4
M	2	2	1	1	6
N	1	1	1	1	4
Urban	J	3	2	3	2	10
Cornwall	Rural	PF	2	2	1	3	8
Urban	PHD	3	2	3	3	11
PHS	1	1	1	1	4
SC	3	2	2	3	10
Total			23	18	17	19	77

**Table A3:** GLMM and LMM model selection based on ΔAIC values

Model	ΔAIC
**Appearance at tables GLMM, *N*=399**	
Appears∼Condition+Corvid+Corvid*Condition+Date+Time+Urban+Condition*Urban+Region+Year	0.0
Appears∼Condition+Corvid+Corvid*Condition+Date+Time+Urban+Condition*Urban+Region	−0.5
Appears∼Condition+Corvid+Corvid*Condition+Date+Time+Urban+Condition*Urban	−0.9
Appears∼Condition+Corvid+Corvid*Condition+Date+Time+Urban	−1.2
Appears∼Condition+Corvid+Corvid*Condition+Date+Time	−1.9
Appears∼Condition+Corvid+Corvid*Condition+Date	+1.4
**Feeding rate, LMM, *N*=176**	
log(Feed.rate)∼Condition+Corvid+Corvid*Condition+Date+Year+Urban+Condition*Urban+Time+Region	0.0
log(Feed.rate)∼Condition+Corvid+Corvid*Condition+Date+Year+Urban+Condition*Urban+Time	−1.91
log(Feed.rate)∼Condition+Corvid+Corvid*Condition+Date+Year+Urban+Condition*Urban	−1.97
log(Feed.rate)∼Condition+Corvid+Corvid*Condition+Date+Year+Urban	−3.91
log(Feed.rate)∼Condition+Corvid+Corvid*Condition+Date+Year	−0.02
log(Feed.rate)∼Condition+Corvid+Corvid*Condition+Date	+0.86
**Visit rate, LMM, *N*=178**	
log(Visit.rate)∼Condition+Corvid+Corvid*Condition+Date+Year+Urban+Condition*Urban+Time+Region	0.0
log(Visit.rate)∼Condition+Corvid+Corvid*Condition+Date+Year+Urban+Condition*Urban+Time	−0.91
log(Visit.rate)∼Condition+Corvid+Corvid*Condition+Date+Year+Urban+Condition*Urban	−0.0
log(Visit.rate)∼Condition+Corvid+Corvid*Condition+Date+Year+Urban	−4.13
log(Visit.rate)∼Condition+Corvid+Corvid*Condition+Date+Year	+1.24
log(Visit.rate)∼Condition+Corvid+Corvid*Condition+Date	+0.97

All models include Trial, Species and Table as random effects. Final models are marked in bold. Dropping any terms listed in final models results in an >2 increase in AIC. An asterisk denotes an interaction term.

**Table A4:** Final GLMM and LMM models for the restricted data set

	Estimate±SE	*z*	*P*
**Appearance at tables GLMM, *N*=225**			
Corvid	2.81±1.16	2.41	0.016
Date	0.03±0.01	3.13	0.002
Litter*Corvid	−3.97±1.06	−3.73	<0.001
Natural*Corvid	−2.71±1.07	−2.54	0.011
Novel*Corvid	−2.61±1.04	−2.51	0.012
**Visit rate LMM, *N*=114**			
Corvid	2.25±0.46	4.88	<0.001
Date	0.01±0.01	2.67	0.008
Litter*Corvid	−0.58±0.44	−1.33	0.183
Natural*Corvid	0.018±0.43	0.04	0.967
Novel*Corvid	−1.36±0.40	−3.40	<0.001
**Feeding rate LMM, *N*=114**			
Corvid	3.06±0.41	7.44	<0.001
Litter*Corvid	−0.37±0.46	−0.80	0.423
Natural*Corvid	0.19±0.46	0.41	0.684
Novel*Corvid	−1.13±0.43	−2.63	0.008

Data contained only the two species from each group that visited the most (jackdaws/rooks and blue tits/great tits). The control condition served as the reference category for all object conditions, noncorvids for species group and rural areas for habitat type. An asterisk denotes an interaction term.

**Table A5:** Cox proportional hazards model on arrival times for the restricted data set

	rho	χ^2^	*P*
Corvid	0.195	6.30	0.012
Corv_before	0.174	7.61	0.006
Litter*Corvid	−0.188	5.69	0.017
Natural*Corvid	−0.071	1.56	0.211
Novel*Corvid	0.010	0.01	0.910
Litter*Urban	−0.232	9.05	0.003
Novel*Urban	0.019	0.118	0.731

Cox proportional hazards model, *N* = 225 observations, 140 events. Data contained only the two species from each group that visited the most (jackdaws/rooks and blue tits/great tits). The control condition served as the reference category for all object conditions, noncorvids for species group and rural areas for habitat type. An asterisk denotes an interaction term.

**Table A6:** Cox proportional hazards models for latency to arrive at tables

Variable	Full model			Minimal model		
rho	χ^2^	*P*	rho	χ^2^	*P*
Date	−0.014	0.073	0.787			
Noncorv_before	−0.106	3.659	0.058			
Year	−0.094	3.284	0.070			
Condition*Species group						
Litter*Corvid	−0.061	0.947	0.330			
Natural*Corvid	−0.066	1.682	0.195			
Novel*Corvid	−0.021	0.084	0.771			
Condition						
Litter	0.026	0.172	0.679	0.065	0.817	0.366
Natural	−0.112	5.889	**0.015**	−0.167	9.891	**0.002**
Novel	0.029	0.157	0.692	0.028	0.143	0.705
Corvid	0.214	9.477	**0.002**	0.192	12.546	**<0.001**
Urban	−0.048	0.544	0.461	−0.085	2.085	0.149
Cornwall	0.103	4.346	**0.037**	0.116	5.412	**0.020**
Corv_before	0.176	8.068	**0.005**	0.256	16.900	**<0.001**
Time	−0.195	12.357	**<0.001**	−0.168	7.607	**0.006**
Condition*Habitat						
Litter*Urban	−0.215	16.422	**<0.001**	−0.243	21.609	**<0.001**
Natural*Urban	0.068	2.158	0.142	0.045	0.703	0.402
Novel*Urban	−0.067	1.070	0.301	−0.055	0.637	0.425

Noncorv_before is a measure of whether a noncorvid species arrived before the current observation. Corv_before denotes whether a corvid species had arrived beforehand. Significant *P* values (*P* < 0.05) are highlighted in bold. The control condition was the reference category for all object conditions, rural areas for the urban gradient and Cambridgeshire for the region.

**Table 1:** Percentage of impervious surface area within the 1 km^2^ grid surrounding the feeding table

Feeding table ID	Region	Classification	Impervious surface area
PH-S, PH-D	Cornwall	Urban	55.25
J	Cambridgeshire	Urban	51.14
SC	Cornwall	Urban	20.87
M, H	Cambridgeshire	Rural	5.7
PF	Cornwall	Rural	3.56
I, K, N	Cambridgeshire	Rural	2.15
B, D	Cambridgeshire	Rural	4.1

Calculated with Google Earth Pro.

**Table 2:** Cox proportional hazards models for latency to arrive at tables

Variable	Minimal model		
rho	χ^2^	*P*
Condition			
Litter	0.065	0.82	0.366
Natural	−0.167	9.89	**0.002**
Novel	0.028	0.14	0.705
Species group (Corvid)	0.192	12.55	**<0.001**
Habitat (Urban)	−0.085	02.09	0.149
Region (Cornwall)	0.116	5.41	**0.020**
Corv_before	0.237	14.37	**<0.001**
Time	−0.168	7.61	**0.006**
Condition*Habitat			
Litter*Urban	−0.243	21.61	**<0.001**
Natural*Urban	0.045	0.70	0.402
Novel*Urban	−0.055	0.64	0.425

Corv_before denotes whether a corvid species had arrived beforehand. Significant *P* values (*P* < 0.05) are highlighted in bold. The control condition was the reference category for all object conditions, rural areas for the urban gradient and Cambridgeshire for the region. See Appendix Table A6 for model with nonsignificant terms.

## Appendix

### Colour analysis

To determine whether the materials used for making novel objects were visually distinct to the birds, we measured their spectral qualities using an Ocean Optics USB2000 spectrometer, with illumination provided by a PX-2 pulsed Xenon lamp. The probe tip was housed in a hollow sheath so that samples were measured at 45 degrees to normal, and we used a Spectralon 99% white reflectance standard (Labsphere) and a dark current reading to standardize scans. Each material was measured three times, each at a different location. Colour distances between material types were calculated using the coldist function of the pavo package in R (Maia, Eliason, Bitton, Doucet & Shawkey, 2013; R Core Team, 2015), using common starlings, *Sturnus vulgaris*, as the visual model. No two materials that contrasted by less than one just noticeable difference (JND) were used on the same object.

### Ringed birds analysis

A total of 76 ringed individuals (67 jackdaws, eight rooks, one jay) participated alongside the many unringed birds, and we analysed the behaviour of this subset of the data. This data set was biased (e.g. a large majority of ringed birds were in Cornwall, only one urban table was sampled and only 23 trials saw ringed visitors), so results must be interpreted with caution. Nevertheless, we ran a similar model to the one in the main text on individuals' appearance during trials, with individual as an additional random effect. We found support for the main conclusion that corvids are neophobic, as individuals were less likely to appear at tables when there was a novel object on the table (GLMM: *N* = 522, estimate + SE = −2.62 + 1.03, *z* = −2.54, *P* = 0.011). Moreover, survival analyses confirmed that the birds were quicker to arrive around litter in urban areas (Cox proportional hazards model, *N* = 522 observations, 109 events, rho = −0.233, χ^2^ = 5.87, *P* = 0.015). We were unable to run formal models on individuals' visit and feeding rates because few individuals visited more than once during a given trial (*N* = 31), and therefore models would have been overparametrized, with the four random effects and even one main effect of experimental condition.

In addition to these analyses, we also looked at the relationship between ringed and unringed visitors to determine how well ringed corvids represented unringed ones. In the areas where we had ringed populations, we found that we could identify a statistically similar percentage of visitors during all conditions (chi-square test, number of visits by ringed birds versus number of visits by unringed birds: χ^2^_3_ = 6.3065, *P* = 0.098). Therefore the ratios of ringed to unringed birds were stable across conditions, and ringed bird behaviour probably predicted unringed bird behaviour. This means that the objects were as likely to be novel for unringed birds as they were for ringed individuals. Additionally, it is unlikely that a small number of unringed birds determined all of the results, otherwise we would have seen particular ringed individuals biasing the results too.
